# Facilitating giant panda crossings of national highway in Wolong area of Giant Panda National Park amid human activities

**DOI:** 10.1002/ece3.70067

**Published:** 2024-07-29

**Authors:** Hu Zhang, Zongkun Shi, Bin Feng, Ying Liu, Zhuo Tang, Xin Dong, Xiaodong Gu, Dunwu Qi, Weihua Xu, Caiquan Zhou, Jindong Zhang

**Affiliations:** ^1^ Key Laboratory of Southwest China Wildlife Resources Conservation (Ministry of Education) China West Normal University Nanchong China; ^2^ School of Ecology and Environment Tibet University Lhasa China; ^3^ Wolong National Nature Reserve Administration Wenchuan China; ^4^ College of Environmental Science and Engineering China West Normal University Nanchong China; ^5^ Forestry and Grassland Administration of Sichuan Province & Sichuan Giant Panda National Park Administration Chengdu China; ^6^ Chengdu Research Base of Giant Panda Breeding Chengdu China; ^7^ Research Center for Eco‐Environmental Sciences, Chinese Academy of Sciences Beijing China

**Keywords:** giant panda, habitat, human activities, nature reserve, road, wildlife corridor

## Abstract

As human activities continue to expand, wildlife persistence faces escalating threats from roads. In Wolong area of Giant Panda National Park, the local giant pandas (*Ailuropoda melanoleuca*) are divided into two population groups along the National Highway G350 (NHG). Therefore, selecting suitable areas to help those giant pandas communicate across the NHG is necessary. In this research, we evaluated the presence of human activities and simulated their absence to analyze how they affect the giant panda's habitat in Wolong. Subsequently, based on the kernel density estimation (KDE) for giant pandas and the main human distribution locations, we selected suitable areas for the population link between the two road sections on the NHG. We simulated the absence of human activities on the two road sections to compare changes in the habitat suitability index (HSI) and connectivity value (CV) relative to their presence. We aimed to carefully select the area for future giant panda corridor plans and simulate whether eliminating human activities will significantly improve the HSI and CV of the area. Our results show that: (1) Human activities presence has led to subtle changes in the landscape pattern of suitable habitats and a decrease in Wolong by 78.76 km^2^ compared to their absence. (2) Human activities presence significantly reduced HSI and CV in the 1000 m buffer along the NHG compared to their absence. (3) The HSI and CV of the 1000 m buffer in the simulated absence of human activities for the two road sections were significantly higher than their presence. This research identified the optimal road section for crossing the NHG to link giant panda population groups and habitats in Wolong. These insights are significant for formulating conservation decisions and corridor plans and for promoting wildlife conservation in reserves amid high levels of human activity.

## Graphical abstract

In the Wolong area of the Giant Panda National Park, giant pandas are divided into two population groups along a national highway. In this research, after considering the main locations of humans and giant pandas, we selected and compared the habitat suitability and connectivity benefits of two key road sections on the national highway. Finally, we identified an optimal road section for the future corridor plan that facilitates the crossing of giant pandas across the national highway in Wolong.

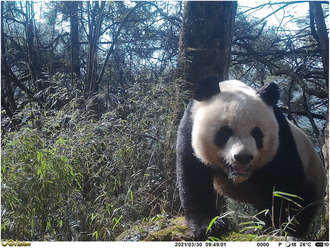

## 1 INTRODUCTION

The relentless intensification of human activities has fundamentally altered the distribution of wildlife globally (Gaynor et al., 2018). Human activities drive habitat reduction and fragmentation, and even cause species extinction in extreme cases (Ceballos et al., 2015; Cooke et al., 2023; Smith et al., 2023). Therefore, investing in biological conservation is essential to maintain and enhance habitat suitability and connectivity for wildlife. However, balancing the costs of management strategies and the ecological benefits poses a significant challenge for contemporary decision‐makers. Thus, researching strategies to balance human activities with wildlife conservation is essential for sustainable development.

**FIGURE 1:**
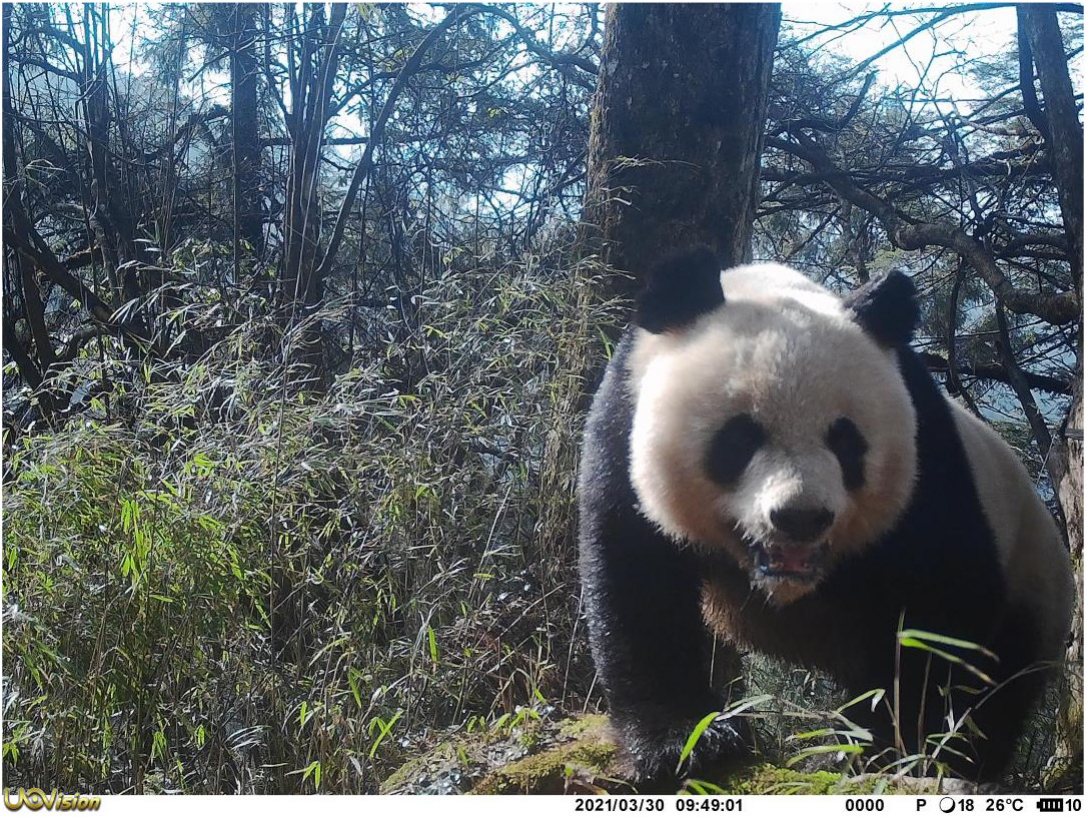
Giant panda (photographed by an infrared camera in Wolong area of Giant Panda National Park).

Research into the impact of roads on wildlife survival and the development of corresponding conservation strategies has been a prevalent topic. Roads cause habitat fragmentation, contribute to traffic‐related wildlife mortality, and impede regular interspecies communication (Lesbarreres & Fahrig, 2012; Trombulak & Frissell, 2000). Those threats are also observed in the giant panda (*Ailuropoda melanoleuca*; Figure 1) (Hu et al., 1985; Kang, 2022; SFD, 2015). Therefore, constructing wildlife corridors in areas heavily impacted by roads is important for preserving ecological balance. Currently, the approach to mitigating the impact of road obstructions through corridors includes the implementation of tunnels, green bridges, and fauna culverts (Reck et al., 2019). Selecting suitable areas for these measures is important to promoting a friendly coexistence between humans and wildlife (Lesbarreres & Fahrig, 2012).

Giant pandas are currently found exclusively in China (Hu et al., 1985; Wei, 2018). Following the fourth National Giant Panda Survey, the conservation status of the giant panda was downgraded from “Endangered” to “Vulnerable” (IUCN, 2016; NFGA, 2021). However, this reassessment masks the ongoing challenges of smaller and more isolated giant panda population groups (Kong et al., 2021). These groups continue to face challenges like habitat fragmentation and the diminution of genetic diversity (SFD, 2015). In response to these challenges, researchers shed light on their struggles and designed a range of solutions, including addressing issues like the loss of habitat connectivity, isolation, and population segmentation (Hu et al., 2017; Li et al., 2023; Wei, 2018; Zhu et al., 2011). These research efforts have led to proposals such as the reconfiguration of the reserve's management zones, implementation of livestock grazing prohibitions, and construction of corridors (Hull et al., 2011; Qiu et al., 2019; Wang et al., 2021; Xu et al., 2006, 2019; Yang et al., 2023; Zhang et al., 2017). Selecting suitable areas for corridor construction is important to strengthen strategies for giant panda conservation, and the importance for corridor design is the analysis of habitat suitability and connectivity (Cushman et al., 2013; Lesbarreres & Fahrig, 2012; Tewksbury et al., 2002; Wang et al., 2014).

Roads as a serious obstacle to the movement of giant panda are currently being widely researched. Although there is no direct evidence, compared to most other bear species, giant pandas are more sensitive to human activities, especially showing a stronger avoidance of roads (e.g., Donatelli et al., 2022; Kang, 2022; Kautz et al., 2021). For example, past research has documented smaller densities and fewer signs of giant pandas further from roads, especially the national highways that cross giant panda reserves (He et al., 2019; Qin et al., 2022; SFD, 2015). Therefore, it is a lengthy process for giant pandas to transition from avoiding roads to approaching roads and achieving migration. Currently, there are few instances of actual construction of corridors over roads for giant pandas, but there have been successful cases. For example, after more than 10 years of effort, giant pandas were recently recorded for the first time using a corridor above a tunnel across National Highway G108 in Zhouzhi National Nature Reserve (CNR, 2023). These pioneering cases make it possible for other areas to develop corridors across roads for giant pandas. Thus, given the current status of many populations of giant pandas being separated by roads, it is necessary to continue researching cross‐road corridors (SFD, 2015; Wei, 2018).

**FIGURE 2:**
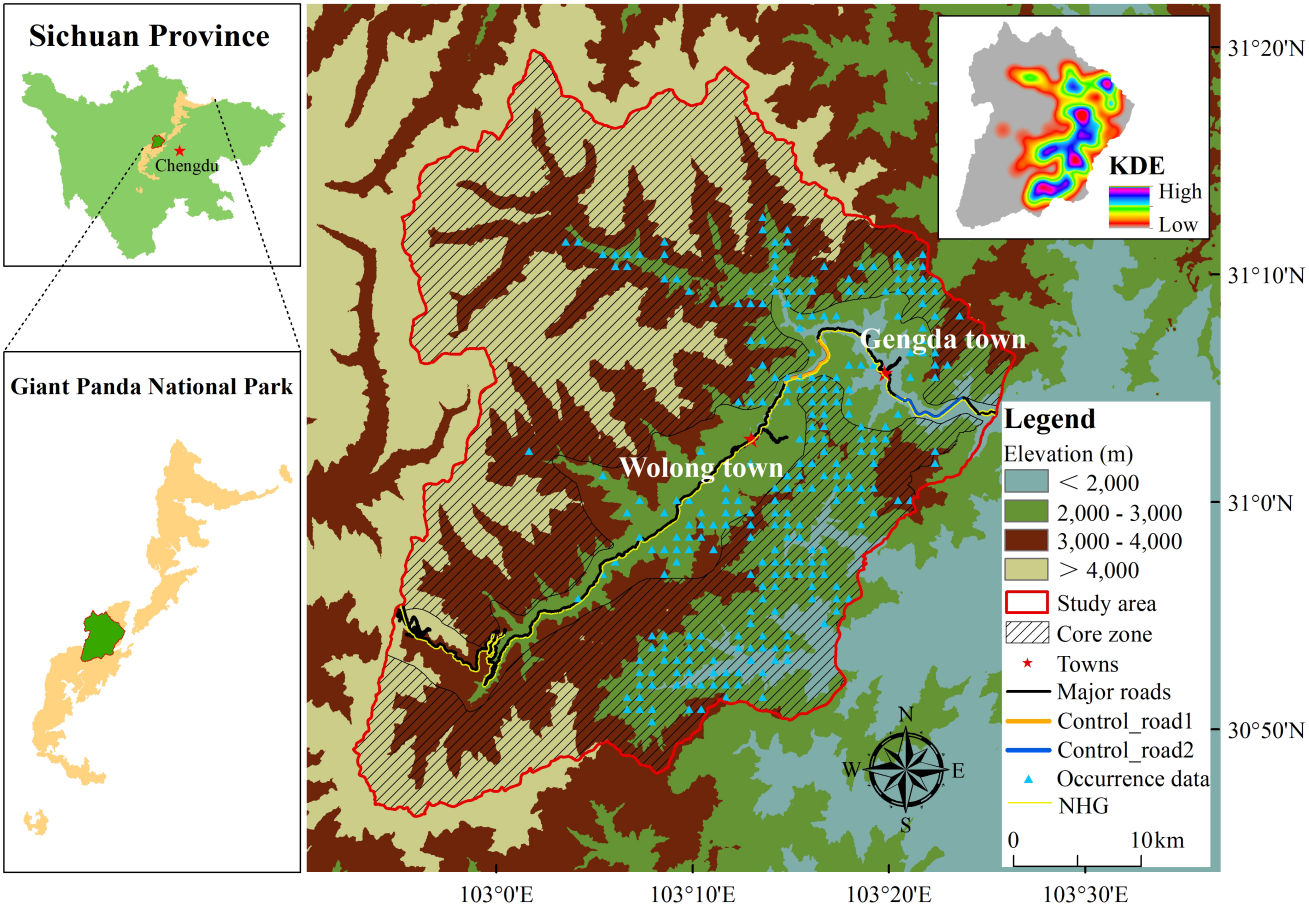
Map of the Wolong in GPNP, Sichuan. The map displays the distribution of the core zone (zone needing maximum protection from the management department), the major roads, and the two towns. Additionally, it delineates the locations of the two road sections, the giant pandas occurrence data, and the map of KDE for giant pandas.

Wolong area of Giant Panda National Park (GPNP) (previous name: Wolong National Nature Reserve) (hereafter referred to as Wolong), designated as a World Natural Heritage Site (WNHS) in 2006, is an internationally renowned giant panda reserve (Hu et al., 1985; NFGA, 2019; WHC, 2006). According to historical records since 1985, the number of giant pandas in Wolong has shown considerable fluctuations, ranging from 72 (Second National Giant Panda Survey, survey period: 1985–1988) initially to 143 (Third National Giant Panda Survey, survey period: 1999–2003), subsequently decreasing to 104 (Fourth National Giant Panda Survey, survey period: 2011–2014), and the last is 142 as defined by Qiao et al. (2019) (SFD, 2015). Over the past decade, the giant pandas in Wolong concentrated in more suitable areas of their habitats because the marginal habitat areas have become more vulnerable due to natural disasters and human activities (Bai et al., 2017). Currently, Wolong is a popular tourist destination in GPNP (He et al., 2021). It encompasses two major towns, Gengda and Wolong town, home to approximately 5000 residents (He et al., 2021). Agriculture, tourist economy, and livestock grazing remain important income sources for the local residents (He et al., 2021; Hull et al., 2015; Wang et al., 2019). Therefore, Wolong contends with continued human activities, even under strict conservation policies (Bai et al., 2020; He et al., 2021). Compounding these challenges is the growing influx of tourists and intense vehicular traffic on National Highway G350 (NHG), which traverses almost the entire Wolong (Figure 2; He et al., 2021). The Fourth National Giant Panda Survey showed how these activities have fragmented the giant panda population into two groups (the two groups are called Wolong‐Caopo and Xiling Snow Mountain‐Jiajin Mountain) along the NHG (NFGA, 2021; SFD, 2015). Furthermore, a notable finding by Qiao et al. (2019) from a 2016 survey revealed that merely 1.4% of individual giant pandas crossed the NHG, typically returning to their original habitat in a short distance after crossing the road into the adjacent habitat. According to past reports, the NHG was upgraded continuously after the Wenchuan earthquake. In 2020, the local government department plans again to invest 71.37 million yuan to upgrade the NHG (SWNNRA, 2020). However, there is still a lack of research on corridor plans for giant pandas across the NHG based on human activities. Therefore, the need remains to develop effective strategies that select the suitable area to construct the corridor to balance human activities with giant pandas conservation in Wolong, particularly in measuring and alleviating migratory impediments and connectivity challenges posed by the NHG.

Here, we simulated the absence of human activities to assess their impact on giant panda habitats in Wolong, with a particular focus on habitat suitability and connectivity around the NHG. Then, based on the kernel density estimation (KDE) for giant pandas and the main human distribution locations, we selected the road sections on the NHG for the link between the two giant panda population groups. We simulated the absence of human activities on those road sections to compare the changes in habitat suitability and connectivity with and without human presence. This research highlights the impact of human activities on giant pandas and assesses whether eliminating human activities would significantly improve the habitat suitability and connectivity of those selected road sections. We aim to carefully select the area with the greatest positive impact on both giant panda conservation and human development for Wolong's future corridor plan. This research will offer valuable insights into facilitating wildlife crossings over large roads amid human activities.

## 2 METHODS

### 2.1 Study area and occurrence data

Wolong area of Giant Panda National Park (120°52′‐103°24′ E, 30°45′‐31°25′ N) was established in 1963 and encompassed a span of 2000 km^2^ in 1975 (Figure 2). The topography predominantly consists of lofty mountains and deep valleys (Hu et al., 1985).

To delineate giant panda habitats, we utilized occurrence data from the Fourth National Giant Panda Survey and research by Qiao et al. (2019) (SFD, 2015; NFGA, 2021). These two giant panda surveys cover almost all areas where giant pandas may appear in Wolong. Meanwhile, to define as complete a range of activities as possible, we also combined the occurrence data of giant pandas collected from our surveys in Wolong in recent years. We reduced redundancy and mitigated spatial autocorrelation by filtering occurrence data in a 1 km grid using the *ENMTools* package (https://CRAN.R‐project.org/src/contrib/Archive/ENMTools) (Warren et al., 2021). This process kept 263 unique occurrence data points for further analysis.

### 2.2 Research scenarios

Apart from the normal circumstances (NC) characterized by the presence of persistent human activities, we propose two hypotheses. Hypothesis 1 (H1) posits that human activities are absent in Wolong (i.e., no residents, roads, and livestock grazing). This hypothesis aims to simulate the giant pandas' habitat under purely natural conditions and assess the impact of human activities. Hypothesis 2 (H2) assumes the absence of human activities in only two control road sections on NHG, selected based on the KDE of giant pandas and the main human distribution locations (Figure 2). Our purpose is to select road sections away from major human activities areas, aiming at the areas with the greatest potential for enhancing giant panda population connectivity. The two control road sections are named Control Road 1 (CR1) and Control Road 2 (CR2), and both are about 6 km in length (Figure 2). Finally, we have three scenarios (i.e., NC, H1, and H2), each representing different human activities presence.

### 2.3 Habitat suitability

We employed the MaxEnt 3.4.1 species distribution model for estimating the habitat suitability for giant pandas, which primarily bases the model on sample locations and environmental factors (Phillips et al., 2006). The model result is a map of the probability distribution for species, which also serves as the habitat suitability index (HSI), ranging from 0 (unsuitability) to 1 (most suitability). In alignment with the preceding research, we acknowledged that environmental factors such as livestock grazing, road, bamboo distribution, and topography influence giant panda habitat selection (Bai et al., 2020; Hu et al., 1985; Ouyang et al., 2001). The Pearson correlation coefficient matrix was used to exclude highly correlated factors to prevent excessive collinearity (|r| ≥0.85). Finally, we obtained nine factors, including Climate (Annual precipitation) + Topography [Digital Elevation Model (DEM), Aspect, Slope] + Vegetation [Normalized Difference Vegetation Index (NDVI), Bamboo Distribution] + Human Activities (Human Pressure, Livestock Grazing) (Table 1). The bamboo distribution and livestock grazing points were extracted from the Fourth National Giant Panda Survey (SFD, 2015; NFGA, 2021). Furthermore, we also used data obtained during surveys in Wolong to compare the bamboo distribution and livestock grazing distribution points to ensure the range was as accurate as possible. The human pressure factor is formed by integrating road points and residential points. Road points are derived from placing points every 1000 m on major roads (Figure 2). The residential points are derived from the GPS data we collected from Wolong in 2016, and also through data we obtained during surveys in Wolong. In addition, we used Google Earth to align the residential points. We resampled each factor layer at a 30 m resolution. Specifically, NC and H2 retain all nine factors, but the control road sections under H2 exclude road points, and H1 retains the remaining eight factors except human activities.

**TABLE 1:** Environment factors for the MaxEnt model.

Environmental factors		Source	Type of variable
Climate	Annual precipitation	https://www.geodata.cn	Continuous
Topography	DEM	https://www.gscloud.cn	Continuous
	Aspect		Continuous
	Slope		Continuous
Vegetation	NDVI	https://www.igsnrr.ac.cn	Continuous
	Bamboo distribution	Euclidean distance from grid to bamboo distribution points is calculated by ArcGIS	Continuous
Land cover	Land cover	https://zenodo.org/records/8176941	Categorical
Human activities	Human pressure	Euclidean distance from grid to human pressure points is calculated by ArcGIS	Continuous
	Livestock grazing	Euclidean distance from grid to livestock grazing points is calculated by ArcGIS	Continuous

The accuracy of the MaxEnt model was ascertained utilizing the receiver operating characteristic (ROC) area under the curve (i.e., AUC) obtained through 10 rounds of Bootstrap method analysis (Phillips et al., 2017), in which 75% of the giant panda occurrence data points were selected as the training data and the remaining 25% as the testing data. The AUC value is assessed by the following criteria: 0.5–0.6 failure; 0.6–0.7 poor; 0.7–0.8 general; 0.8–0.9 good; and 0.9–1.0 excellent (Araujo et al., 2005). We employed maximum training sensitivity plus specificity Cloglog threshold to divide HSI, resulting in suitable and unsuitable habitats (Phillips et al., 2017).

### 2.4 Suitable habitat fragmentation

We employed the landscape pattern index to evaluate the fragmentation status of suitable habitats for giant pandas. The indices utilized include the patch density index (PD), largest patch index (LPI), contagion index (CONTAG), and splitting index (SPLIT). We used the *Landscapemetrics* (https://CRAN.R‐project.org/web/packages/landscapemetrics) package for analytical purposes (Hesselbarth et al., 2019).

### 2.5 Habitat connectivity

Habitat connectivity is important for evaluating wildlife dispersal potential between distinct source locations (McRae et al., 2008). We employed the Circuitscape 5 model to analyze the habitat connectivity value (CV) for giant pandas. The Circuitscape 5 model is based on circuit theory and integration in the Julia programming language (Anantharaman et al., 2019). The run of the Circuitscape 5 model relies on the resistance surface and source locations. We calculated the inverse of the HSI by utilizing geometric calculation tools in ArcGIS 10.8, generating a resistance surface. We determined that the giant panda occurrence data serve as source locations. This model culminated in acquiring detailed insights regarding the probability of giant pandas dispersal across various grid trajectories. In the resulting connectivity layer, we determined each grid's corresponding likelihood of movement as the CV, where a larger value indicates stronger connectivity.

### 2.6 Comparison of effects of different scenarios

Based on the ArcGIS 10.8 spatial analysis tools, we analyzed the suitable habitat alterations in areas and fragmentation compared to the three scenarios. Furthermore, we conducted two comparative analyses to reveal the impact of H1 and H2 on HSI and CV. First, we compared the NC with H1, focusing on HSI and CV changes in a 1000 m buffer around the NHG. We employed a non‐parametric paired‐sample *T*‐test to analyze the difference. Second, we compared NC and H2, replicating the same buffer zone on controlled road sections. The same approach was used to evaluate the difference in HSI and CV. Then, we performed a non‐parametric *T*‐test on HSI and CV between CR1 and CR2 under the H2 to ascertain the most important control road section for prioritization. This buffer size refers to findings by Ouyang et al. (2001) regarding the typical range size of giant pandas affected by roads in Wolong. All statistical analyses were conducted using the Wilcox.test function in R 4.2.1.

## 3 RESULTS

### 3.1 Suitable habitat and fragmentation

**FIGURE 3:**
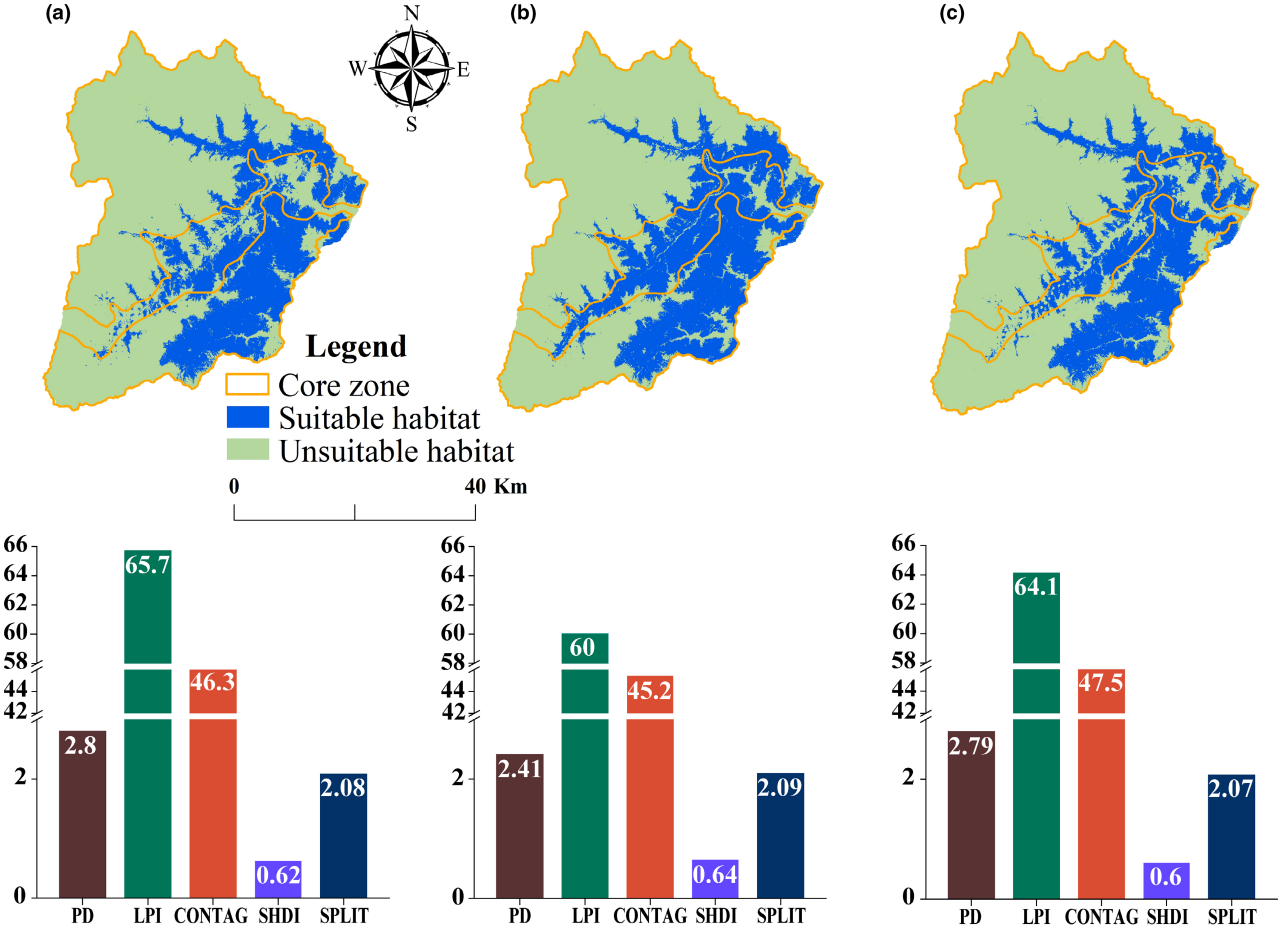
Map of suitable habitats for giant pandas in Wolong under the three scenarios: (a) NC; (b) H1; and (c) H2.

The MaxEnt models developed showed good performance in the three scenarios. The model for NC resulted in an average AUC of 0.877 ± 0.005 (mean ± SD), with a category‐suitable habitat threshold of 0.36. This model estimated 620.73 km^2^ of suitable habitat, constituting roughly 31.04% of Wolong, of which 450.78 km^2^ lay in the core zone (Figure 3a). For H1, an average AUC of 0.854 ± 0.008, with a suitable habitat threshold of 0.38. It estimated 699.49 km^2^ of suitable habitat, representing about 34.97% of Wolong, with 499.92 km^2^ in the core zone (Figure 3b). For H2, an average AUC of 0.869 ± 0.010, with a suitable habitat threshold of 0.43. This model estimated 587.04 km^2^ of suitable habitat, approximately 29.35% of Wolong, including 412.54 km^2^ in the core zone (Figure 3c).

The landscape pattern index analyses revealed the fluctuations in suitable habitat landscape indices across the three scenarios, but they were insignificant (Figure 3).

### 3.2 Habitat connectivity

**FIGURE 4:**
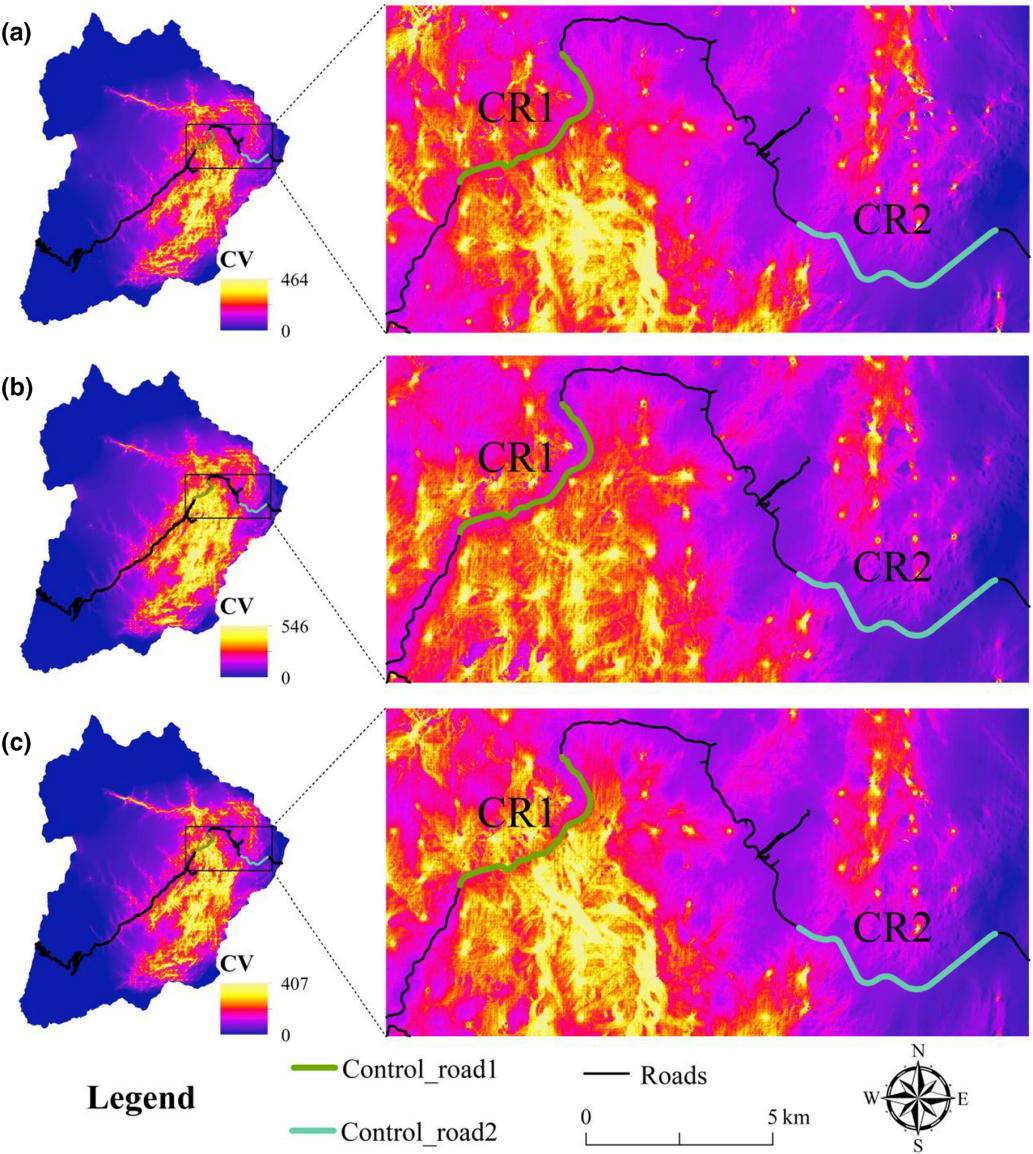
Map of habitat connectivity for giant pandas in Wolong under the three scenarios: (a) NC; (b) H1; and (c) H2.

Habitat connectivity showed greater robustness in the southeast across all three scenarios and exhibited varying degrees of change near the control road sections (Figure 4).

### 3.3 Effects for habitat and road from different scenarios

Compared to H1, amid the influence of human activities presence (i.e., NC scenario), the suitable habitat for giant pandas has contracted by 78.76 km^2^, with a reduction of 49.14 km^2^ in the core zone. Compared to NC, H2 led to an additional reduction of suitable habitat by 33.69 km^2^ and a decrease of 38.24 km^2^ in the core zone. Fragmentation in giant panda habitats exhibited subtle fluctuation across three scenarios, with the largest change observed in the LPI (Figure 3).

**FIGURE 5:**
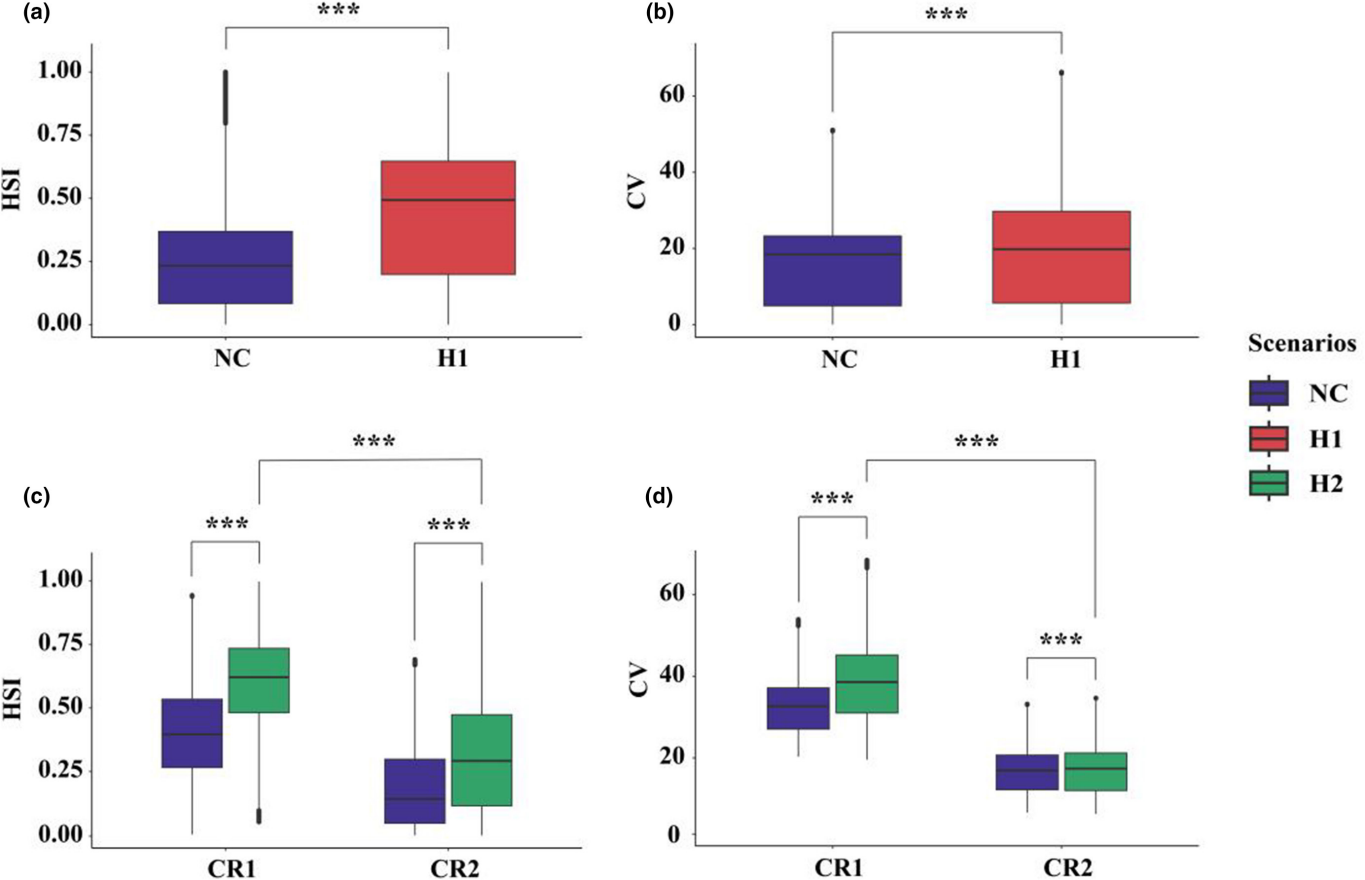
T‐test analyses were conducted for HSI and CV across three scenarios, with means replacing redundant outliers. Analyses included: (a,b), comparing HSI and CV between NC and H1 in a 1000 m buffer of NHG. (c,d), Comparing HSI and CV between NC and H2 in a 1000 m buffer of control road sections, and considering the difference of CR1 and CR2 under H2. *** Represents high significance *p* < .001.

T‐test revealed that the presence of human activities (i.e., NC scenario) significantly lowered the HSI and CV compared to H1 (Figure 5a,b). HSI and CV were significantly higher under H2 than NC (Figure 5c,d). CR1's HSI and CV were significantly higher than those of CR2 under H2 (Figure 5c,d).

## 4 DISCUSSION

Research on wildlife habitat suitability and connectivity is imperative, as both are intricately linked to human activities (Bastille‐Rousseau & Wittemyer, 2021; Dickson et al., 2019; Li et al., 2019). Our findings show that human activities have not intensified the fragmentation of suitable habitats for giant pandas in Wolong, but they compressed 78.76 km^2^ of the available suitable habitat. Furthermore, comparing the NC and H1, it is evident that human activities have constricted suitable habitats into two isolated patches, which seem segregated along the NHG. This pattern of habitat distribution suggests that human activities have intensified the fragmentation into islands of suitable habitats for giant pandas in Wolong, aligning with findings from other researchers (Liu et al., 2001; Ouyang et al., 2001; SFD, 2015). Additionally, in a 1000 m buffer at the NHG, human activities significantly lower HSI and CV, which may also be additional evidence of the divergence of the giant panda population in Wolong. Past research on human activities has primarily focused on livestock grazing and management zoning, often overlooking the specific quantification of the isolation effect of roads on giant pandas in Wolong (Bai et al., 2020; Hull et al., 2015; Wang et al., 2019; Zhang et al., 2014). Our conservation approach gravitated toward a select road section for the corridor plan, facilitating a link for crossing the NHG among giant panda's local populations and habitats.

Roads undoubtedly disrupt wildlife habitats and migratory routes, as evidenced by numerous researches (Lesbarreres & Fahrig, 2012; Trombulak & Frissell, 2000). Our findings under H2 revealed a decrease in giant panda suitable habitats after implementing controlled road sections, compared to NC. However, *T*‐tests on HSI and CV in H2's control road sections revealed them to be significantly higher than NC. Like He et al. (2019) and Qin et al. (2022) research conclusions, our findings also suggest that giant pandas tend to avoid roads, but eliminating human activities in controlled road sections will also improve habitat suitability and connectivity. This may encourage giant pandas to move to more suitable habitats, rather than forcing them toward less suitable edge areas due to human activities. Our results seem to support the opinion from Lesbarreres and Fahrig (2012), significantly increased the habitat suitability and connectivity in the control road sections, even amid the influence of human activities, indicating the feasibility of the H2 and future giant panda corridor construction.

A multitude of research has underscored diverse conflicts between human development and wildlife conservation, sparking extensive research on balancing wildlife conservation and human development (Pimm et al., 2014; Pooley et al., 2021; Tian et al., 2019; Yin et al., 2021). Commonly proposed policies include enforcing restrictions on livestock grazing and planting in reserves and limiting residential expansion. However, these measures are costly and may even impact the livelihood of residents and economic and regional development in Wolong. The 2019 policy announcement by the Chinese government to establish the GPNP in 2021 exemplifies an approach that considers both human development and wildlife conservation (NFGA, 2019; Xu et al., 2019). Therefore, we intend to complement the giant panda conservation and development strategy rather than impose a suggestion for a restrictive policy. We advocate the construction of a giant panda corridor at CR1 to mitigate the impact of human activities and the NHG, which is a positive way to promote the coexistence of humans and giant pandas.

In our research, the control road sections we selected were functionally analogous to tunnels or green bridges, effectively alleviating the impact of roads on wildlife by preserving adjacent habitats. However, in the context of designing corridors for giant pandas, tunnels have often been overlooked by researchers, although they are over hundreds of kilometers and have potentially positive effects (Chen et al., 2021; Gracanin & Mikac, 2023; Sijtsma et al., 2020). Our research under the NC highlights this insufficiency. Conversely, the H2 demonstrates their potential to enhance giant panda population connectivity. We advocate considering the inclusion of tunnels in connectivity and corridor construction for wildlife populations. Reevaluating the impact of roads on wildlife through effective tunnel utilization may mitigate the risks associated with corridor plans, reduce costs, and expedite the process (Jia et al., 2023; Wang et al., 2014). Therefore, we recommend managing CR1 based on existing tunnels to address connectivity issues for the two local giant panda populations amid pressure from human development in Wolong.

**FIGURE 6:**
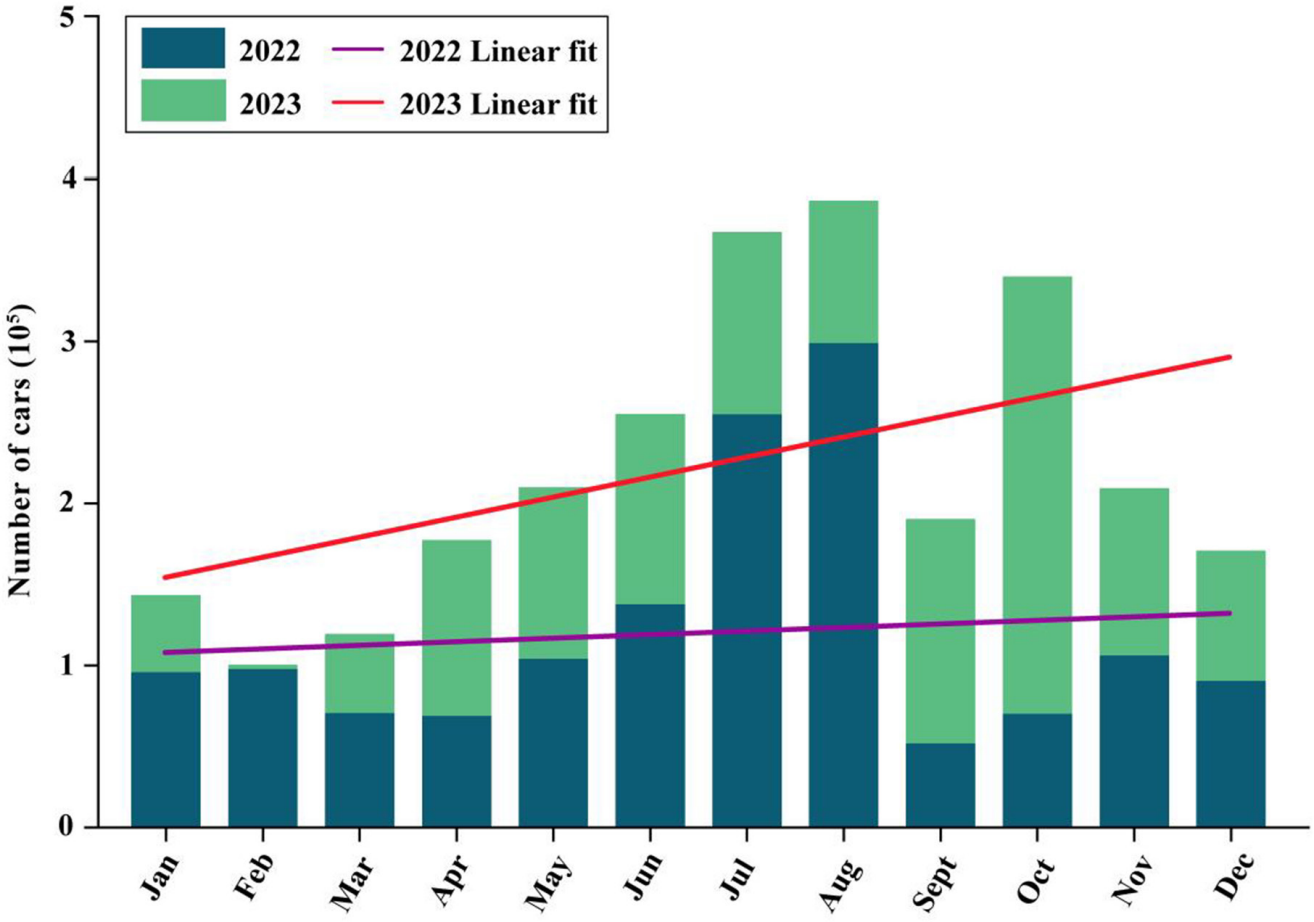
Wolong's monthly vehicle flow statistics on National Highway G350 for the years 2022–2023, including the linear fit curve for the 2‐year period (data from Wolong National Nature Reserve Administration).

**FIGURE 7:**
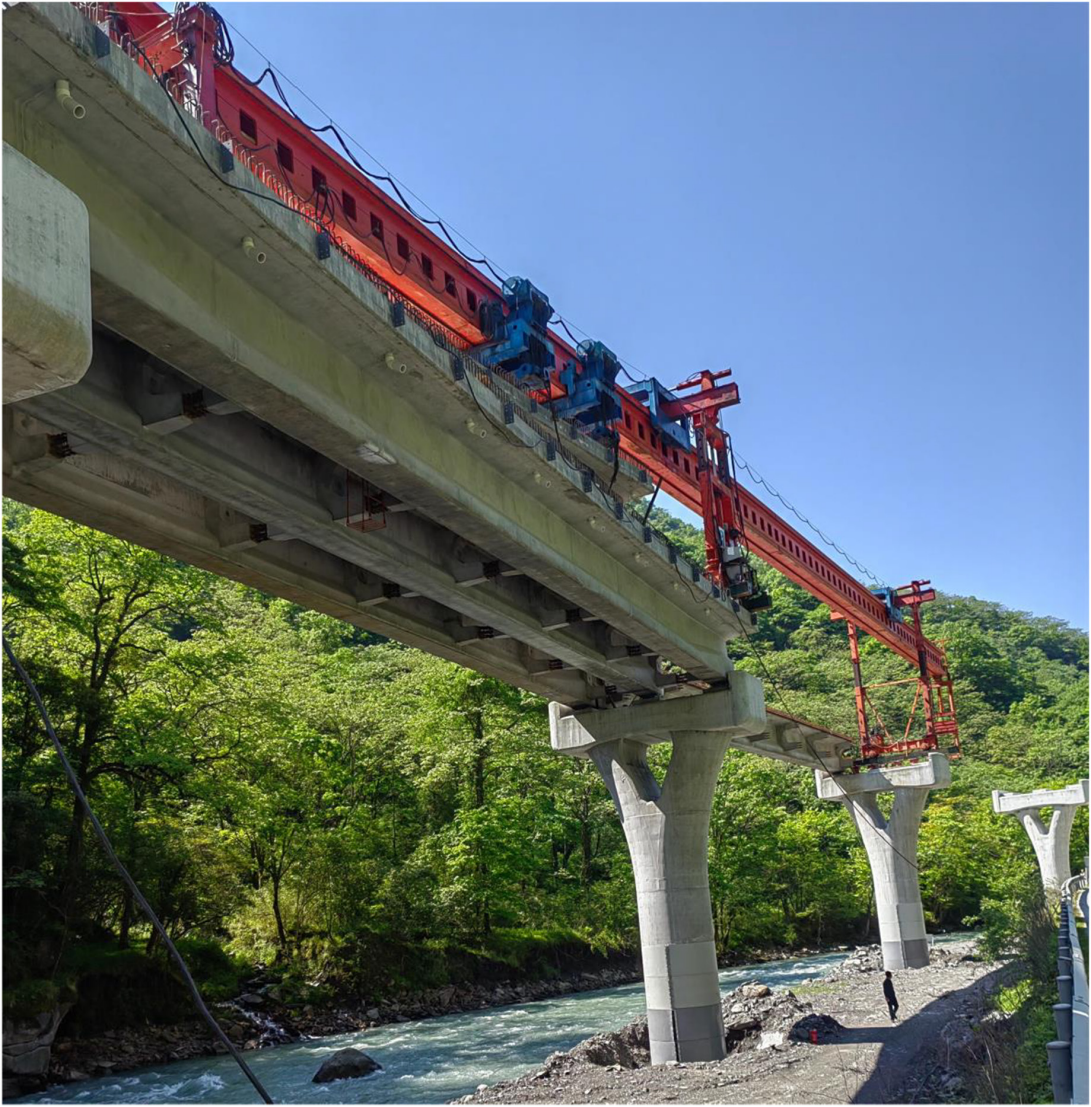
The sightseeing railway is under construction in Wolong (shooting time May 2024).

Our research has successfully evaluated the impact of giant panda habitats in Wolong amid human activities. Although methods for selecting corridor locations differ, similar to our conclusion, some research supports the placement of a corridor near CR1 (e.g., Wang et al., 2021; Xu et al., 2006). But compared to other research, we further demonstrate the effectiveness of implementing the H2 scenario to enhance the habitat suitability and connectivity of local giant panda populations in a key road section on the NHG. These results provided a more in‐depth theoretical basis for the construction of the giant panda corridor in Wolong. However, it is important to acknowledge that our research may underestimate the extent of human activities, considering Wolong's popularity and the significant year‐on‐year surge in vehicle flow on the NHG (the number of cars increased by 84.93% year on year from 2022 to 2023; Figure 6) (He et al., 2021; Wang et al., 2019). Meanwhile, a sightseeing railway is also being constructed in Wolong extending along NHG (Figure 7). Furthermore, we have recognized that the corridor construction area identified in Wolong from our research overlaps with the core conservation zone of GPNP in some parts. Indeed, the core conservation zone set by the GPNP means stricter conservation. But to support the opinions of Wang et al. (2021) and Yang et al. (2023), some appropriate solutions are still being sought in GPNP by the Chinese government for the management of human activities in different areas and the coordination of their development relationships with giant pandas' conservation. Therefore, we suggest that the cross‐road corridor plans and construction are still important content for Wolong and the recently established GPNP in the future. However, as a complex work, giant panda corridor construction still needs to fully address the practical challenges of habitat corridor construction, such as sources of funding and public attitudes (Kang & Li, 2016). Thus, determining the ideal extent of the road sections in Wolong requires a more comprehensive evaluation, which also aids in selecting the appropriate form of the corridor plan. Future research can explore the deep details of giant panda corridor construction and connectivity solutions for other wildlife species in Wolong so that the corridor construction can benefit multiple species and enhance comprehensive ecological connectivity.

## AUTHOR CONTRIBUTIONS

**Hu Zhang:** Conceptualization (lead); formal analysis (lead); investigation (equal); visualization (lead); writing – original draft (lead). **Zongkun Shi:** Methodology (supporting). **Bin Feng:** Formal analysis (supporting). **Ying Liu:** Methodology (supporting); visualization (supporting). **Zhuo Tang:** Investigation (supporting); resources (supporting). **Xin Dong:** Writing – review and editing (supporting). **Xiaodong Gu:** Resources (equal). **Dunwu Qi:** Resources (equal); supervision (equal). **Weihua Xu:** Resources (equal); supervision (equal). **Caiquan Zhou:** Resources (equal). **Jindong Zhang:** Conceptualization (equal); funding acquisition (lead); project administration (lead); supervision (lead); writing – review and editing (lead).

## FUNDING INFORMATION

The key project of the State Forestry and Grassland Administration “Study on Key Technologies for Conservation of Wild Giant Panda Populations and Its Habitats within Giant Panda National Park System” (grant number: CGF2024001); National Natural Science Foundation of China (grant number: U21A20193; #42071279; and #32270551).

## CONFLICT OF INTEREST STATEMENT

The authors declare no conflict of interest.

## Data Availability

Related data are openly available in Dryad at: https://doi.org/10.5061/dryad.kh18932fm
